# Simultaneous Infrared Observations of the Jovian Auroral Ionosphere and Thermosphere

**DOI:** 10.1029/2024JA032891

**Published:** 2024-11-30

**Authors:** Ruoyan Wang, Tom S. Stallard, Henrik Melin, Kevin H. Baines, Luke Moore, James O’Donoghue, Rosie E. Johnson, Emma M. Thomas, Katie L. Knowles, Paola I. Tiranti, Steve Miller

**Affiliations:** ^1^ School of Physics and Astronomy University of Leicester Leicester UK; ^2^ Department of Mathematics Physics and Electrical Engineering Northumbria University Newcastle upon Tyne UK; ^3^ Jet Propulsion Laboratory California Institute of Technology Pasadena CA USA; ^4^ Space Science and Engineering Center University of Wisconsin‐Madison Madison WI USA; ^5^ Center for Space Physics Boston University Boston MA USA; ^6^ Department of Meteorology University of Reading Reading UK; ^7^ Department of Physics Aberystwyth University Aberystwyth UK; ^8^ Department of Physics and Astronomy University College London London UK

**Keywords:** Jupiter, ionosphere, thermosphere, aurora, temperature, infrared

## Abstract

Simultaneous observations of $H_{3}^{+}$ and $H_{2}$ in Jupiter's northern infrared aurora were conducted on 02 June 2017 using Keck‐NIRSPEC to produce polar projection maps of $H_{3}^{+}$ radiance, rotational temperature, column density, and $H_{2}$ radiance. The temperature variations within the auroral region are ∼700−1000 K, generally consistent with previous studies, albeit with some structural differences. Known auroral heating sources including particle precipitation, Joule heating, and ion drag have been examined by studying the correlations between each derived quantity, yet no single dominant mechanism can be identified as the main driver for the energetics in Jupiter's northern auroral region. It appears that a complex interaction exists between the heating driven by various mechanisms and the cooling from the $H_{3}^{+}$ thermostat effect. Comparisons between the $H_{3}^{+}$ temperature and the line‐of‐sight ion velocity in the reference frame of (a) the planetary rotation and (b) the neutral atmosphere further suggest that the local thermodynamic equilibrium effect may play an important role in thermospheric heating at Jupiter. Along with previously reported heating events that occurred in both the lower and upper atmosphere, it is speculated that the heating source may originate from an altitude above Jupiter's stratosphere but below the peak altitude of $H_{3}^{+}$ overtone and $H_{2}$ quadrupole emissions.

## Key Points

- Scanning maps of $H_{3}^{+}$ radiance, temperature, column density, and $H_{2}$ radiance are produced using Keck‐NIRSPEC
- Correlations between derived quantities are investigated to study the heating mechanism of Jupiter's auroral thermosphere
- The source of heating may originate from an altitude between the stratosphere and $H_{3}^{+}$ overtone and $H_{2}$ emission peak altitudes

## 1 Introduction

Since the discovery of $H_{3}^{+}$ on Jupiter (Drossart et al., 1989), it has long been used to probe the upper atmospheric chemistry, energetics, and dynamics of giant planets, as well as the magnetosphere‐ionosphere‐thermosphere coupling processes. Searching for temperature variations in $H_{3}^{+}$ has been one of the main focuses of spectroscopic studies (see the review by Miller et al., 2020, and references therein). Despite many attempts over the years, mechanisms behind the heating and cooling of the Jovian thermosphere remain ambiguous. The interaction between Jupiter's upper atmosphere and magnetosphere, through energetic particle precipitations (Grodent et al., 2001; Yelle & Miller, 2004), Joule heating (Millward et al., 2005; Smith et al., 2005), and ion drag (Miller et al., 2000; Millward et al., 2005; Smith et al., 2005), has been thought to be one of the major causes of thermospheric heating at Jupiter. Joule heating refers to the thermalized kinetic energy that heats the atmosphere, and ion drag is the result of an exchange of kinetic energy between neutral and ionized gases, both of which stem from relative motions between the two components (Smith et al., 2005; Vasyliūnas & Song, 2005). Measurements of $H_{3}^{+}$ temperature can be used to infer the thermospheric temperature, as these ions are considered to be in quasi‐local thermodynamic equilibrium local thermodynamic equilibrium (LTE) with the surrounding neutral atmosphere (e.g., Melin et al., 2005; Miller et al., 2000; Tao et al., 2011). Spectroscopic infrared observations of Jupiter's aurora have reported $H_{3}^{+}$ ion velocity, temperature, density, and emission intensities (e.g., Chaufray et al., 2011; Johnson et al., 2017, 2018; Moore et al., 2017; Raynaud et al., 2004; Stallard et al., 2001, 2002). Recent works using instruments with higher spatial and spectral resolution have produced auroral, polar, and even global mappings (e.g., Adriani et al., 2017; Moore et al., 2017; O'Donoghue et al., 2021). An approach to investigate particle precipitation, Joule heating, and ion drag as potential heating mechanisms is thus to compare the physical quantities measured directly from the observed $H_{3}^{+}$ emissions (Johnson et al., 2018; Raynaud et al., 2004).

Direct measurements of $H_{2}$ emission intensity, temperature, and column density allow investigations of the physical and chemical mechanisms that cause the structural changes and dynamics of Jupiter's thermosphere. The near‐infrared provides a unique window for investigating auroral emissions at high latitudes. Within the atmospheric K‐band window (∼1.9−2.5μm), several prominent $H_{2}$ quadrupole lines and multiple $H_{3}^{+}$ overtone lines span, suggesting the viability of probing infrared auroral emissions from both neutrals and plasmas using K‐band spectroscopy. Theoretically, it is possible to perform analogous studies on the infrared quadrupole lines of $H_{2}$ as on $H_{3}^{+}$ emission lines (Trafton et al., 1989), but such studies have been very limited in the past. Kim et al. (1990) measured the rotational temperature of ∼ 540–1,230 K from $H_{2}$ $S_{1}$(0), $S_{1}$(1), and $S_{1}$(2) lines in the southern aurora with notable large error bars. The main challenge of data processing is the relatively low signal‐to‐noise ratio, primarily due to uncertainties associated with instrumental effects and background noise.

Raynaud et al. (2004) observed the auroral emission of Jupiter near 2.1 μm and managed to capture the $H_{2}$ $S_{1}$(1) quadrupole line with several $H_{3}^{+}$ overtone lines, which allow them to map the emission distribution of $H_{2}$. The thermospheric temperature and density could not be measured due to insufficient $H_{2}$ lines. With the same data, Chaufray et al. (2011) determined the thermospheric wind velocity of <1.0 km/s; only the upper limit can be constrained due to large uncertainties. Wang et al. (2023) conducted another simultaneous observation in the K band and observed the $H_{3}^{+}$ and $H_{2}$ emission lines, from which the authors derived the line‐of‐sight Doppler shift velocities of ion and neutral winds. Their result revealed subcorotational neutral velocities within the auroral region, showing that Jupiter's thermosphere departs from corotating with the internal rotation of the planet. Consequently, it is important to account for the neutral components when examining the correlations between thermospheric temperature and ion wind velocities. Previous measurements of vertical distributions have found that the peak $H_{3}^{+}$ overtone and $H_{2}$ quadrupole emissions share the same altitudinal range at ∼700−900 km in the Jovian upper atmosphere (Kita et al., 2018; Uno et al., 2014). Under these circumstances, Wang et al. (2023) calculated the relative velocity between $H_{2}$ and $H_{3}^{+}$, that is, the ion wind in the context of the neutral atmosphere, defined as the effective ion drift. With the thermospheric wind velocity considered, the effective ion drift may be a more accurate indicator of ion‐neutral interactions in Jupiter's upper atmosphere.

We present here spatial maps of $H_{3}^{+}$ radiance, temperature, and column density, along with the $H_{2}$ radiance, obtained from a high‐resolution simultaneous observation of Jupiter's northern aurora. On top of this, we analyze the correlations within these parameters and between the effective ion drift derived in Wang et al. (2023) to investigate the heating mechanism of the auroral thermosphere. Details of the observation and data reduction are described in Section 2. Polar maps of the ion and neutral parameters are provided in Section 3. The results are discussed in Section 4, followed by the conclusion in Section 5.

## 2 Observation and Data Processing

The observation was conducted on 02 June 2017 from 06:23 to 07:40 UTC, using the NIRSPEC instrument on the Keck II telescope. Jupiter's northern aurora was scanned twice from the pole toward the equator with an east‐west slit of size $0.288\times 24^{\prime \prime }$ and a step size of $0.2^{\prime \prime }$. The wavelength range of the instrument is set to the K band, aiming at ∼2.062−2.416μm in the near‐infrared, allowing for simultaneous observations of $H_{2}$ quadrupole and $H_{3}^{+}$ overtone emission lines. Positions of the echelle and the cross‐disperser were set to 63.9 and 35.6, respectively. The weather was clear and stable throughout the night, with the average air mass measured at ∼ 1.1 and the seeing recorded as $∼0.6^{\prime \prime }$. Jupiter's equatorial diameter at the time of observation was $∼40.6^{\prime \prime }$, with a sub‐Earth latitude of ∼−3.098 degrees. A total of 38 spectral images, excluding calibration exposures and sky frames, were obtained by combining six integrations, each 10 s long. Table 1 summarizes the $H_{3}^{+}$ overtone and $H_{2}$ quadrupole emission lines used for analysis, all from echelle order 32 (2.382–2.416 μm) and order 36 (2.117–2.148 μm).

**Table 1:** Spectroscopic Properties of $H_{3}^{+}$ and $H_{2}$ Lines Used for Analysis

Order	Assign.	λ (μm)	$\nu_{2}$	$J_{upper}$	$\omega_{upper}$ (${\text{cm}}^{-1}$)	ω (${\text{cm}}^{-1}$)	$A_{if}$ ($s^{-1}$)	$g_{ns}$
36	Q(5,2)	2.1221	2–0	5	5835.27	4712.29	67.04	2
36	P(4,1)	2.1277	2–0	3	5469.61	4700.15	56.44	2
36	Q(4,2)	2.1314	2–0	4	5396.33	4691.98	58.93	2
36	R(8,8)	2.1343	2–0	9	6269.9	4686.76	161.8	8/3
36	Q(3,2)	2.1380	2–0	3	5041.16	4677.27	42.06	2
36	P(3,0)	2.1439	2–0	2	5117.06	4664.3	63.76	4
36	$S_{1}$(1)	2.1220	1–0	3	6951.3	4712.9	3.47	21
32	$Q_{1}{\left(1\right)}^{*}$	2.4066	1–0	1	6149.0	4155.25	4.294	9
32	$Q_{1}{\left(2\right)}^{*}$	2.4134	1–0	2	6471.4	4143.5	3.051	5

*Note.* The lines with an asterisk are associated with large uncertainties (discussed in detail in the Results section). References of the line list are Neale et al. (1996) and Roueff et al. (2019).

**Figure 1:**
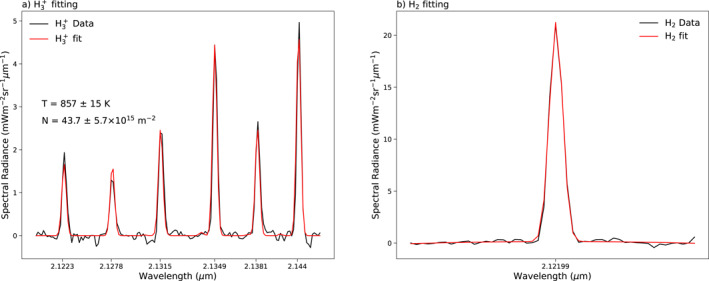
Example observed (a) $H_{3}^{+}$ and (b) $H_{2}$ spectra (black) with model fit (red). $H_{3}^{+}$ temperature and column density are retrieved with uncertainties.

An open‐source Python package h3ppy is used to derive the $H_{3}^{+}$ radiance, rotational temperature, and column density. Figure 1 shows an example of the observed spectra and model fits, attached with the derived rotational temperature and column density. Gaussian fits are performed to the $H_{2}$ spectra as in Wang et al. (2023) to retrieve the $H_{2}$ radiance. The resultant scanning maps are polar projected into jovigraphic coordinates by identifying the slit position relative to the locations of the limb of Jupiter in the slit‐viewing camera image. The planetary limb can be determined using the Jovian polar flattening, the sub‐Earth latitude of the telescope, and the central meridian longitude. The calculated limb is then used to assign latitude and longitude to each spatial and spectral pixel onto a 360×180 degree grid with a bin size of 1°. The limb brightening effect is removed from calculations of radiance and column density (correction not needed for temperature due to the same variation in the radiance used for calculation, under the assumption of uniformity), following the same method as in Johnson et al. (2017).

## 3 Results

**Figure 2:**
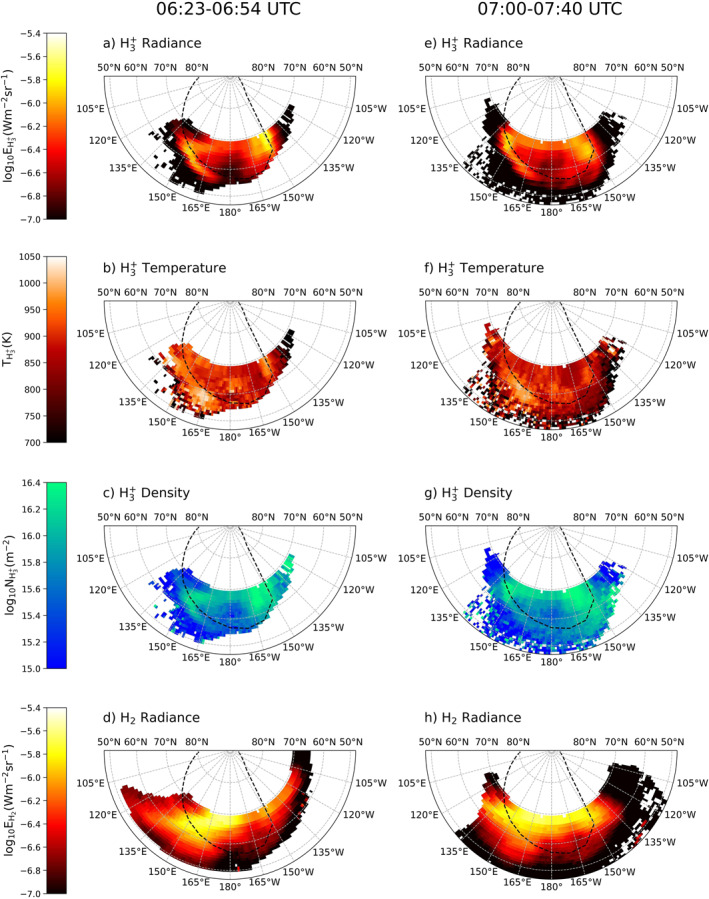
Polar projected maps of the measured $H_{3}^{+}$ (a, e) radiance, (b, f) temperature, and (c, g) column density, along with the $H_{2}$ (d, h) radiance. The time span of each scan taken on 02 June 2017 is shown at the top. The dashed black line marks the main emission of Jupiter's aurora, obtained from the model of Grodent et al. (2001).

Figure 2 shows the main results of this study: scanning maps of $H_{3}^{+}$ radiance, rotational temperature, column density, and $H_{2}$ radiance. Figures 2a–2d refer to the first scan and Figures 2e–2h to the second scan, with the latter covering a larger region toward the middle latitude. The $H_{3}^{+}$ radiance shown in Figures 2a and 2e have the mean values of 0.36 and 0.28 $\mu {\text{Wm}}^{-2}{\text{sr}}^{-1}$, respectively, which are of the same order of magnitude as the $H_{3}^{+}$ overtone profiles in Kita et al. (2018). The main emission is sharp on the dawn side and more expanded on the dusk side, bright along the auroral peak of the Grodent et al. (2001) model in both sectors. Additionally, a large dark spot is seen in the polar region at ∼60°N, 180°. These features broadly agree with the projected radiance map of $H_{3}^{+}$ overtone emissions in Raynaud et al. (2004) and fundamental emissions in Moore et al. (2017) and Johnson et al. (2018).

The derived $H_{3}^{+}$ rotational temperatures shown in Figures 2b and 2f range approximately between 700 and 1,000 K, with mean values of 892 and 849 K, which are in agreement with previous measurements. Along the auroral peaks, two high‐temperature regions are found near 60°N, 165°E and 65°N, 150°W, along with a large cold spot near 65°N, 165°W. Raynaud et al. (2004) and Moore et al. (2017) reported similar temperature variations, though these structures appear to be highly versatile. We also identify a large area with lower temperature poleward of the main emission in the dawn polar region, which has been previously seen in Johnson et al. (2018).

The column densities of $H_{3}^{+}$ are shown in Figures 2c and 2g, with mean values of 8.05 and $7.97\times 10^{15}\;m^{-2}$. Large densities are found along the two auroral peaks and in the polar region just below 70°N, whereas the region around ∼65°N, 180° is notably less dense than the surroundings. The high‐density region on the dusk side is expanded, with the peak offsets ∼5° poleward of the dusk main emission. On the dawn side, the dense region becomes narrow and confined. Again, these results are similar to Moore et al. (2017), where the densities are found to be higher in the dusk polar region and along the dawn auroral peak. Johnson et al. (2018) also reported similar morphology, but their peak densities in the dusk sector aligned more closely to the main emission without clear offsets. The error bars of $H_{3}^{+}$ column densities in Raynaud et al. (2004) were too large to make a comparison. Note that an anticorrelation relationship exists between density and temperature, which has been previously reported in Melin et al. (2014).

$H_{2}$ radiance shown in Figures 2d and 2h are averaged to 0.51 and 0.42 $\mu {\text{Wm}}^{-2}{\text{sr}}^{-1}$, respectively. Regions with high radiance are found on the dawn side and across the polar region above 60°N, extended to the dusk side. Below 60°N, from ∼170°E to ∼160°W, the radiance significantly drops below the average, resulting in notable dark regions in both scans. Raynaud et al. (2004) derived the $H_{2}$ radiance from the same $S_{1}$(1) line and also identified a similar bright region in the dawn sector of the northern hemisphere. Considering that the two observations were conducted with different instruments in a nearly two‐decade gap, it might be possible that this large bright spot is due to a persistent phenomenon in the thermosphere. However, more studies involving polar mapping of directly measured $H_{2}$ radiance are needed to validate our speculation.

We have attempted to determine the rotational temperature and column density from the observed $H_{2}$ lines $S_{1}$(1), $Q_{1}$(1), and $Q_{2}$(2), following the same method applied to the $H_{3}^{+}$ lines. However, $Q_{1}$(1) cannot be separated from the adjacent absorption line and skyline, while $Q_{2}$(2) lacks a sufficient S/N ratio. We cannot confirm whether the quantities derived using these two lines represent real thermospheric conditions or refer to any artifacts of the fitting process. $H_{2}$ temperature and density are not shown due to these concerns. Only $S_{1}$(1) is available to derive the $H_{2}$ wind velocity (Wang et al., 2023) and radiance (Figures 2d and 2h).

**Figure 3:**
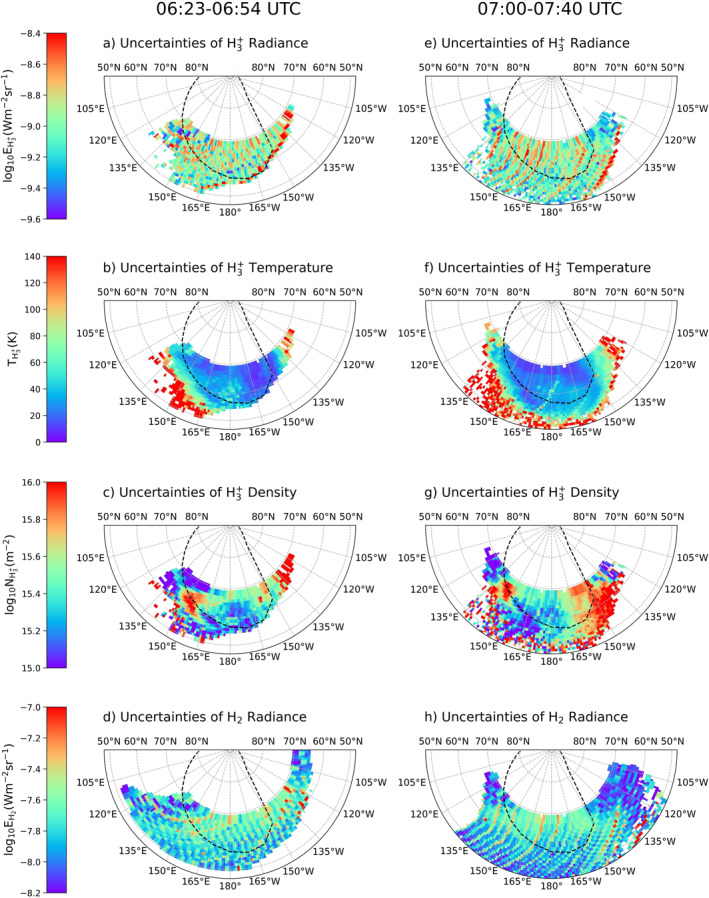
Uncertainties corresponding to the measured $H_{3}^{+}$ (a, e) radiance, (b, f) temperature, (c, g) column density, and $H_{2}$ (d, h) radiance. The plotting format is identical to Figure 2, except for the color bars.

The uncertainties of the measured quantities are mainly associated with instrumental effects, the signal‐to‐noise ratio of the spectra, and the fitting errors propagated throughout the calculations. Figure 3 shows the corresponding uncertainties of the polar projection maps. From top to bottom of the uncertainty map: Figures 3a and 3e for $H_{3}^{+}$ radiance, Figures 3b and 3f for $H_{3}^{+}$ temperature, Figures 3c and 3g for $H_{3}^{+}$ density, and Figures 3d and 3h for $H_{2}$ radiance. Variations in the uncertainties of $H_{3}^{+}$ and $H_{2}$ radiance depend on the quality of the data and the background noise. These are generally consistent throughout the map after data reduction and removal of the continuum disk. Regions poleward of the main auroral emission are associated with lower uncertainties for $H_{3}^{+}$ temperature, where the signals are significantly stronger than those of the subauroral regions. The uncertainty maps of $H_{3}^{+}$ column density for both scans are positively correlated with the main results, where the uncertainties are higher in the region of higher densities due to lower temperature. The $H_{3}^{+}$ radiance is driven both by temperature and density. Although the density is high, the temperature is the main driver of the radiance, keeping the signal‐to‐noise ratio comparatively low. The overall signal‐to‐noise ratio remains relatively consistent due to this reason.

## 4 Discussion

### 4.1 Correlations Between $H_{3}^{+}$ Radiance, Temperature, and Density

**Figure 4:**
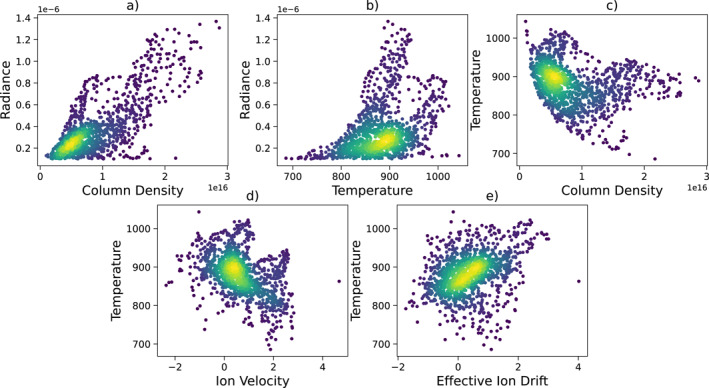
Scatter plots between (a) radiance and column density, (b) radiance and temperature, (c) temperature and column density, (d) temperature and ion wind velocity, and (e) temperature and effective ion drift. The velocities have been reported previously in Wang et al. (2023). A Gaussian kernel‐density estimate is included in the colors to show the probability density of each variable. A lighter color represents a higher probability density, that is, a higher probability of occurrence of the event.

We have determined the correlations between derived $H_{3}^{+}$ parameters to investigate potential connections. Data points used in the calculations only include those with intensities larger than 0.1 $\mu {\text{Wm}}^{-2}{\text{sr}}^{-1}$ to ensure strong S/N ratios. Due to data limitation, the Pearson correlation coefficient can be computed for only the second scan, which yields 0.75 between radiance and column density, 0.38 between radiance and temperature, and −0.28 between column density and temperature. Figure 4 shows the scatter plots between each quantity.

Relatively strong and positive correlations are found between radiance and column density, as shown in Figure 4a. Such relations can also be seen from the morphological comparisons between Figures 2a, 2c and Figures 2e, 2g, showing that regions with high radiance also have high densities, mainly along the auroral peaks and the polar region at 70°N. Positively correlated radiance and density have been reported in previous studies (e.g., Johnson et al., 2018; Moore et al., 2017; Stallard et al., 2002). Changes of $H_{3}^{+}$ radiance could result from variations in temperature, density, or a combination of both. Given that only the correlation between radiance and temperature is relatively lower, the density appears to be the more dominant driver of the emission intensity.

Correlations between the radiance and temperature of $H_{3}^{+}$ are difficult to quantify; Figure 4b reveals an unclear trend. Morphologically, when comparing Figures 2a and 2e with Figures 2b and 2f, regions such as 60°N, 165°E and 70°N, 150°W are found with enhanced temperature and radiance. On the other hand, at regions 65°N, 140°E, 65°N, 180°, and 65°N, 160°W, for example, temperature and radiance appear to be inversely correlated. Stallard et al. (2002) have seen such complex variations in the vibrational temperature derived from $H_{3}^{+}$ fundamental emission lines. Lystrup et al. (2008) later confirmed an anticorrelation between radiance and temperature determined from $H_{3}^{+}$ ion density profiles obtained using Keck‐NIRSPEC.

The lack of correlation between radiance and temperature is not unexpected. Higher temperatures naturally occur higher in the atmosphere. However, the thermostat effect of $H_{3}^{+}$ provides cooling to the auroral region (Miller et al., 2000). At such high altitudes, densities are lower and, therefore, there is less gas, potentially less $H_{3}^{+}$, to radiate heat. As altitude increases, $H_{3}^{+}$ will be less thermally equilibrated, and individual $H_{3}^{+}$ molecule will thus radiate less efficiently (Miller et al., 2013). When a gas emits radiation from a planetary atmosphere, it inevitably produces a cooling effect that counters any warming caused by particle precipitation, EUV heating, chemical reactions, or conductive and mechanical heating, as indicated by Melin et al. (2006). The $H_{3}^{+}$ thermostat effect is most efficient when the gas is in LTE, but continues to function, although less effectively, even when the gas is not thermally equilibrated. At Jupiter, $H_{3}^{+}$ ions were predominantly produced in the main emission regions deeper within the atmosphere at around ∼550 km (Melin et al., 2005). The $H_{3}^{+}$ thermostat effect is more effective in this region, so the temperature will decrease. The peak emission altitude of $H_{3}^{+}$ overtone emissions is at ∼700−900 km (Kita et al., 2018; Uno et al., 2014), but the temperature measured in this work matches previous studies using $H_{3}^{+}$ fundamental lines (Johnson et al., 2018; Moore et al., 2017), which implies that the heating for both layers may be driven by the same mechanism. The temperature of the emitting $H_{3}^{+}$ is affected by its vertical position in the atmosphere, the magnitude of electron precipitation energy, Joule heating, ion drag, and the degree to which the relevant $H_{3}^{+}$ ions are in LTE.

Figure 4c shows that the correlation between column density and temperature is modest and negative. Morphological comparisons between Figures 2b and 2c and 2f‐g reveal that denser regions are generally colder, except near 60°N, 160°E and 65°N, 150°W. Potential anticorrelations between $H_{3}^{+}$ temperature and density have been reported in Lam et al. (1997) and Raynaud et al. (2004), although the uncertainties were too large to distinguish physical and statistical results. Such inverse correlations are expected to be seen in dense regions of $H_{3}^{+}$ with low temperature due to the $H_{3}^{+}$ thermostat effect (Miller et al., 2010). Moore et al. (2017) observed a cooling event of $H_{3}^{+}$ temperature alongside the increase of column density by comparing two different nights of ground‐based observations separated by a 7‐day gap. The temperature and density variations in their study were larger than the associated measurement uncertainties. Melin et al. (2014) has previously reported such an inverse relationship between density and temperature and attributed to an actual physical anticorrelation within Jupiter's atmosphere rather than artifacts of the fitting process. Adriani et al. (2017) showed that the auroral temperatures in the north and south differ significantly in both magnitude and behavior when compared in the local time, yet $H_{3}^{+}$ temperature still tended to be inversely proportional to the column density in the southern aurora. Energetic particle precipitation produces ionization in the upper atmosphere; a higher rate of ionization will produce more $H_{3}^{+}$ and thus increase the column density (Grodent et al., 2001). Johnson et al. (2018) reported a modest positive correlation between temperature and column density by comparing maps of $H_{3}^{+}$ parameters with high spatial resolution, which suggests dominant heating by particle precipitation. In a study of the observed auroral event by Stallard et al. (2001, 2002), Melin et al. (2006) modeled the auroral thermal balance based on observational data and showed that particle precipitation only contributes to a minor extent to the increase in temperature. Particle precipitation is likely to be highly variable in regulating the Jovian thermospheric temperature.

### 4.2 Correlations Between $H_{3}^{+}$ Temperature and Velocity

To investigate the effect of the interaction between Jupiter's upper atmosphere and magnetosphere, Johnson et al. (2018) measured the rotational temperature of $H_{3}^{+}$ and compared with the line‐of‐sight ion velocities in the planetary reference frame derived in Johnson et al. (2017), with the neutral atmosphere assumed in corotation with Jupiter. The authors found that the correlation between $H_{3}^{+}$ velocity and temperature was not strong. For the same comparison, we have transformed the line‐of‐sight $H_{3}^{+}$ ion wind velocity reported in Wang et al. (2023) from the observer reference frame to the planetary rotation frame by subtracting the rotation rate of Jupiter. Assuming that the thermosphere corotates with the planet, we compute a Pearson correlation coefficient of −0.40 between temperature and ion velocity, with a general trend of anticorrelation shown in Figure 4d, which is significantly different from Johnson et al. (2018) (∼0.71). Structures of the ion velocity (Johnson et al., 2017; Wang et al., 2023) and ranges of the rotational temperature (Johnson et al., 2018 and Figures 2d and 2f) in both studies align well with each other and are consistent with past measurements (Chaufray et al., 2011; Moore et al., 2017; Stallard et al., 2001, 2002). The discrepancy may be caused by a departure from LTE due to altitudinal differences between $H_{3}^{+}$ fundamental and overtone emissions.

Giles et al. (2016) simultaneously measured the kinetic, rotational, and vibrational temperature using $H_{3}^{+}$ fundamental and overtone lines in the M band and found different values of each, suggesting a possible LTE breakdown. However, Stallard et al. (2002) reported positive correlations between vibrational temperature and ion velocities using $H_{3}^{+}$ fundamental and hot band emissions, consistent with the positive correlation between rotational temperature and $H_{3}^{+}$ velocities calculated in Johnson et al. (2018). The positive correlations noted in both studies suggest that, for $H_{3}^{+}$ fundamental emissions peaking at ∼550 km, a quasi‐LTE appears to be valid, matching predictions by Melin et al. (2005) that $H_{3}^{+}$ fundamental emissions are least affected by the non‐LTE effect. Tao et al. (2011) showed that the departure from LTE is not significant until above ∼1,000 km. As a result, we consider that our assumption of quasi‐LTE for $H_{3}^{+}$ overtone emissions (∼700−900 km; Uno et al., 2014; Kita et al., 2018) can be deemed reasonable; the difference between results in this study and in Johnson et al. (2018) is likely due to other reasons yet to be known. However, a rigorous study of the LTE effect is suggested to validate its impact at different altitudes in the upper atmospheres of giant planets.

Joule heating is the thermalized kinetic energy heating the atmosphere, resulting from the relative motion between ions and neutrals in the upper atmosphere (Smith et al., 2005). The magnitude of Joule heating is determined by several factors and one of them is the velocity difference between the neutrals and plasmas (small‐scale fluctuations in electric fields may also affect the magnitude of Joule heating; Smith et al., 2005). In this particular case, we expect to see the largest Joule heating in regions where the difference between neutral and ion velocities is the largest. Significantly different spatial correlations in Johnson et al. (2018) and this study indicate that short‐term observations can not reveal the full picture of the dynamical Jovian system with rapidly fluctuating inputs that drive strong temperature gradients.

By taking into account the subcorotational thermospheric wind, as shown in Figure 4e, a weak positive correlation has been found between the effective ion drift in Wang et al. (2023) and the $H_{3}^{+}$ rotational temperature measured in this study, with a Pearson correlation coefficient of 0.32. This is significantly different from the value calculated without considering the neutral motions. Such a correlation relation may be relevant to the ion drag energy in the heating of Jupiter's upper atmosphere. In addition to Joule heating, the relative motion between ions and neutrals in the upper atmosphere can also lead to ion drag, another component of heating due to the exchange of kinetic energy that provides considerable energy inputs to the upper atmosphere (Smith et al., 2005; Vasyliūnas & Song, 2005). However, it is still not yet clear whether these two components should be discussed separately.

Most previous models have treated Joule heating and ion drag as a whole. Smith and Aylward (2009) predicted the occurrence of maximum Joule heating and ion drag at the peak Pedersen conductivity layer, where the ion‐neutral collision frequency equals the ion gyrofrequency; the altitude of this layer has been modeled to peak near 500 km (Tao et al., 2011). Melin et al. (2006) showed that Joule heating and ion drag deposit energy at the altitude of peak $H_{3}^{+}$ ion density, corresponding to the altitude of peak Pedersen conductivity. Calculated based on Tao et al. (2009), Wang et al. (2023) showed that ion drag is only a weak term at depth but dominates the accelerating force compared to all the forces that affect the neutral atmosphere in and above the main ionospheric region (>400 km). The effective ion drifts measured from $H_{3}^{+}$ overtone emissions were predicted to broadly represent the effective ion flows between ∼400−600 km. The overlap altitude at ∼500 km suggests that the impact of Joule heating and ion drag to the temperature variations at the peak layer of $H_{3}^{+}$ fundamental emission are highly likely linked to the neutral thermosphere as predicted. Simulations by Yates et al. (2020) suggested that subcorotating neutral flows would lead to smaller Pedersen currents in the ionosphere and thus decrease Joule heating and ion drag. The weak positive correlation shown in Figure 4e may be an indicator of such a modeled case. Future models are thus suggested to include the effect of neutral thermospheric winds, as well as separate inputs of Joule heating and ion drag energy.

Deep in the lower atmosphere, Sinclair et al. (2017) reported an increase in temperature in the northern auroral region of Jupiter's stratosphere at an altitude corresponding to a pressure of ∼10μbar, below the stratosphere–thermosphere boundary (∼360 km or ∼0.34μbar; Seiff et al., 1998). The authors proposed that the heating may be due to heat conducted from higher thermospheric altitudes, involving the interaction of downward precipitated electrons with molecules in the upper atmosphere, Joule heating, ion drag, and energy released from exothermic chemical reactions of ions. Yates et al. (2020) reported a negative vertical temperature gradient above ∼500 km in their model and argued that this could be due to the model not including energy sources at high altitudes. Although the temperature measured in this study is derived from the $H_{3}^{+}$ overtone emissions located at a much higher altitude (∼700−900 km; Kita et al., 2018; Uno et al., 2014), the value of the temperature is still highly comparable to the results reported in previous studies (Johnson et al., 2018; Moore et al., 2017), which were derived from the $H_{3}^{+}$ fundamental emissions (∼550 km; Melin et al., 2005) Majeed et al. (2009) modeled the vertical profile of heating in Jupiter's auroral region and noticed a heating event dominated by ion drag in the northern auroral oval between 0.2 and 0.01 μbar. These pressures correspond to altitudes higher than the stratosphere–thermosphere boundary. It is possible that the source of thermospheric heating originates from an altitude above the stratosphere but below the peak layer of $H_{3}^{+}$ overtone emissions, releasing heat to the surrounding regions and altitudes above and below. Verification of these speculations would need direct observations of Jupiter's thermosphere. The limitations of ground‐based infrared observations of $H_{2}$ quadrupole emission reported in our study may be overcome using the upgraded Keck‐NIRSPEC (Martin et al., 2018), potentially benefiting future work to decipher the unknown mechanism behind the high Jovian thermospheric temperature.

## 5 Conclusions

We have observed $H_{2}$ quadrupole and $H_{3}^{+}$ overtone emissions simultaneously in Jupiter's northern auroral zone on 02 June 2017 using Keck‐NIRSPEC and present high‐resolution polar maps of $H_{3}^{+}$ radiance, rotational temperature, column density, and $H_{2}$ radiance. Although there are slight structural differences, the derived parameters of $H_{3}^{+}$ and $H_{2}$ share broad similarities with those measured in previous observations Raynaud et al. (2004), Moore et al. (2017), Johnson et al. (2018). We have compared these physical quantities to search for potential correlations with known auroral heating sources such as particle precipitation, Joule heating, and ion drag. Additionally, we have compared the temperature with ion wind velocity in the context of Jupiter's rotation rate and the neutral wind velocity previously reported in Wang et al. (2023). $H_{3}^{+}$ radiance generally reveals a strong correlation with density but is not highly correlated with temperature, while a weak anticorrelation has been found between density and temperature. Between $H_{3}^{+}$ temperature and ion velocity, there is a negative correlation when neutral velocities are not considered and a positive correlation after taking into account the thermospheric wind. It is unclear whether a single dominant heating mechanism is responsible for Jupiter's auroral thermospheric heating. The results suggest a complex interaction between heating by a combination of multiple drivers and cooling due to the $H_{3}^{+}$ thermostat effect. The dependence of the temperature of the emitting $H_{3}^{+}$ on its vertical position in the atmosphere has also been discussed. The level of $H_{3}^{+}$ ions under the LTE effect may be of significance to the thermospheric heating in Jupiter. Comparisons with previously observed auroral heating events deep in Jupiter's stratosphere (Sinclair et al., 2017) and models of vertical temperature profiles (Majeed et al., 2009; Yates et al., 2020) suggest that the heat source may originate from an altitude between the stratosphere and peak layer of $H_{3}^{+}$ overtone and $H_{2}$ quadrupole emissions. Simultaneous observations of both $H_{3}^{+}$ and $H_{2}$ with improved instruments could potentially resolve more details of structure variability, especially with direct measurements of $H_{2}$ thermospheric temperature. With a full investigation of the LTE effect, the driver of heating may be further understood.

## Data Availability

The data used in this study are publicly available at the Keck Observatory Archive (KOA, http://koa.ipac.caltech.edu/cgi‐bin/KOA/nph‐KOAlogin), which is operated by the W. M. Keck Observatory and the NASA Exoplanet Science Institute (NExScI), under contract with the National Aeronautics and Space Administration.
